# Antioxidant Intervention Against Microplastic Hazards

**DOI:** 10.3390/antiox14070797

**Published:** 2025-06-27

**Authors:** Zhihua Wang, Yunting Wang, Jian Zhang, Guoquan Feng, Shuhan Miao, Rongzhu Lu, Xinyu Tian, Yang Ye

**Affiliations:** 1 Department of Gastroenterology, Affiliated Hospital of Jiangsu University, Zhenjiang 212001, China; 1000011866@ujs.edu.cn; 2 Department of Preventive Medicine and Public Health Laboratory Science, School of Medicine, Jiangsu University, Zhenjiang 212013, China; angel_wangyunting@163.com (Y.W.); lurz@ujs.edu.cn (R.L.); 3 Department of Cardiology, Zhenjiang First People’s Hospital, Zhenjiang 212050, China; 15061494388@163.com; 4 Department of Radiology, Affiliated Hospital of Jiangsu University, Zhenjiang 212001, China; fgqtjj@163.com; 5 Department of Health Care, The Fourth Affiliated Hospital of Jiangsu University, Zhenjiang 212013, China; miaoshuhan@126.com; 6 Department of Laboratory Medicine, Nanjing Drum Tower Hospital, Nanjing University Medical School, Nanjing 210008, China; jipinxinyu@163.com

**Keywords:** antioxidant, microplastic, phytochemical

## Abstract

Microplastic pollution (<5 mm) poses a serious threat to the environment and human health, inducing cellular stress damage in organisms (especially through oxidative stress). The damage results from excessive reactive oxygen species and impaired defense mechanisms, affecting energy production, organelles, and triggering inflammation. Antioxidants (such as vitamin C, curcumin, and quercetin) reduce stress markers and inflammation by neutralizing harmful molecules, activating protective pathways, and regulating autophagy, providing potential protection. However, practical applications face challenges such as low absorption rates, large individual variations, and unclear long-term safety. Research needs to delve into the molecular interaction mechanisms, develop effective delivery systems for antioxidant combinations, and formulate evidence-based strategies. Addressing the complexity of microplastics (size, shape, additives) and their cross-ecosystem impacts requires multidisciplinary collaboration. This review explores the oxidative stress mechanisms induced by microplastics, assesses the potential and limitations of antioxidant interventions, and provides a basis for environmental health risk management.

## 1. Introduction

Polyester materials take more than 400 years to completely decompose. The material revolution began with Parke’s celluloid (in 1862), was industrialized by Bakeland’s phenolic resin (in 1907), and experienced explosive growth under the impetus of petrochemical catalytic technology in the 1950s. As of 2023, the cumulative production exceeded 10 billion tons, surpassing the total biomass of all terrestrial vertebrates. This “plastic age” will become a geological marker in future sedimentary rocks in the form of polymer strata [1]. The global annual production has soared from 2 million tons in 1950 to 486 million tons in 2019, with packaging materials accounting for 42% (average lifespan < 1 month), construction materials accounting for 19%, and textiles accounting for 14%. Over 10,000 additives enhance performance but hinder recycling. The core contradiction lies in the fact that 36% are disposable items, and 67% of fishing gear ultimately pollutes the ocean, creating a paradox where production has increased by 200 times while the recycling rate is only 9%. Approximately 79% of discarded plastics accumulate in terrestrial ecosystems, with the density of microplastics in coral reef areas reaching 127,000 particles/km^2^—through tissue wear and toxin release, the disease rate increases by 83%. Atmospheric transport leads to global redistribution: plastic sedimentation in US protected areas amounts to 1320 tons per year (equivalent to 300 million plastic bags). Freshwater systems retain over 90% of river microplastics, becoming permanent reservoirs [2,3]. Plastic pollution has invaded key zones of the Earth: from the sediments of the Mariana Trench to ice core samples from glaciers. The total amount of artificial plastics (8.3 billion tons) exceeds the total dry weight of surface plants. This marks the arrival of the Anthropocene—synthetic polymers have profoundly changed the biogeochemical cycles [4,5].

In recent years, microplastics have emerged as a concerning environmental pollutant, drawing widespread attention, largely due to their presence being detected across ecosystems globally. These particles form through the fragmentation of larger plastic waste materials while also arising from different sources—some being intentionally produced for commercial applications, others resulting when bigger plastic objects deteriorate over time. That means that these pollutants enter environments through multiple pathways [6]. Furthermore, the growing accumulation of microplastics has sparked discussions around potential consequences they might carry for both human health and natural ecosystems, requiring thorough examination to better grasp their long-term implications [7,8].

To reduce the harm of microplastics to the human body, recent studies have proposed strategies to reduce the toxicity of many microplastics. Some researchers suggest using advanced filtration technologies and biodegradable polymer substitutes to reduce the release of microplastics into the environment [9]. It is also necessary to emphasize strengthening industry supervision and environmentally friendly manufacturing to reduce microplastic emissions [10], as well as researching enzyme degradation and biobased materials as sustainable solutions [11]. Some researchers have proposed the possibility of using nanotechnology to enhance wastewater treatment to trap microplastics [12]. In this article, we specifically discuss the harm of microplastics to the human body, their mechanism of action, antioxidants, and effects.

Microplastics can enter the human body through ingestion, inhalation, and dermal contact with the skin [13]. In biological systems, microplastics can induce various toxicological effects, primarily associated with oxidative stress. Oxidative stress is characterized by an imbalance between the production of reactive oxygen species (ROS) and antioxidant defense systems in the body, leading to cellular damage, inflammation, and various diseases. Given the potential health hazards associated with microplastic exposure, there is a growing interest in identifying effective interventions, particularly the use of antioxidants, to counteract the oxidative damage caused by microplastics.

Studies have shown that natural antioxidants alleviate or reverse microplastic-induced oxidative damage through a variety of mechanisms. Most papers hold the view that natural antioxidants have the function of alleviating oxidative damage. For instance, kaempferide significantly alleviates the oxidative damage of polyethylene microplastics on testicular tissue by activating the Nrf-2/Keap-1 pathway, enhancing the activities of superoxide dismutase (SOD) and glutathione peroxidase (GPx), thereby reducing lipid peroxidation and DNA damage [14]. Similarly, cyanidin-3-O-glucoside indirectly reduced oxidative stress levels in mice by promoting intestinal microplastic excretion and regulating intestinal microflora balance while restoring intestinal barrier function [15]. In the human intestinal Caco-2 cell model, nobiletin inhibits the overproduction of reactive oxygen species (ROS) and protects mitochondrial function by inducing autophagy to clear the accumulation of nanoplastics [16]. Additionally, what other ways are there to reduce the bioaccumulation of microplastics in the body? A study on the biodegradability of caterpillar larvae has shown that for microplastics made of polyvinyl chloride (PVC), polystyrene (PS), and polylactic acid (PLA), the biological degradation process can alleviate oxidative damage because it can reduce the long-term accumulation of microplastics [17]. At the same time, there are still different voices that exist in the literature that argue that natural oxides can reverse body damage caused by oxidative stress caused by microplastics. For example, astilbin significantly improved testicular histopathological damage and decreased sperm quality induced by polystyrene microplastics by directly scavenging ROS and inhibiting the expression of inflammatory factors, such as TNF-α and IL-6 [18]. In the Caenorhabditis elegans, exposure to nano-polystyrene and its chemically modified derivatives caused differential oxidative stress responses, suggesting that the intervention strategy of antioxidants needs to be designed according to the specific chemical structure of microplastics [19].

This review aims to elucidate the biological mechanism by which microplastics induce cellular damage in organisms through oxidative stress, evaluate the potential of antioxidant intervention measures (such as vitamin C, curcumin, etc.) in mitigating such damage, and identify the current research gaps in order to provide direction for the formulation of evidence-based environmental health strategies.

Currently, our focus is on microplastics and oxidative stress. According to PubMed and Web of Science, the existing studies mostly single out the literature that focuses on microplastics and the field of antioxidants. However, I believe that integrating the two, such as the reversal or alleviation of oxidative stress caused by microplastics through antioxidants, might be a more worthy research point. Secondly, most of the existing literature only focuses on one mechanism caused by microplastics or an antioxidant. But, in my studies, I have elaborated on most of the existing mechanisms and antioxidants and summarized them together. Moreover, this article conducts a systematic assessment of human evidence for the first time and breaks through by proposing that antioxidant intervention needs to consider dosage, source, and co-exposure factors, providing a theoretical framework for future research design. Finally, this article points out the shortcomings of the existing literature and provides directions for further improvement of future research.

To provide an up-to-date overview of antioxidant intervention against microplastic hazards, a systematic literature search was performed across PubMed and Web of Science for articles published between 1993 and 2025. Key search terms included “Antioxidant”, “Microplastic”, “Phytochemical”, and their combinations. Inclusion criteria focused on peer-reviewed original research and reviews in English. This process yielded over 1000 publications, with 240 central references selected for in-depth analysis and discussion in this review.

## 2. Microplastics: Sources and Impact on Health

### 2.1. Sources of Microplastics

Microplastics are classified into two categories based on their sources as follows:

Primary Microplastics: These small plastic particles are engineered for specific applications, such as microbeads used in personal care products and industrial abrasives.

Secondary Microplastics: These result from the mechanical breakdown of larger plastic products due to environmental factors such as UV radiation, mechanical abrasion, and biological degradation.

The widespread use of plastics and inadequate waste management practices have resulted in significant accumulation of microplastics in both terrestrial and aquatic environments, contributing to global pollution issues [20,21]. Microplastics can enter the human body mostly through three routes: inhalation, ingestion, and cutaneous. Atmospheric microplastics (MPs) and atmospheric nanoplastics (NPs) may be found deep in human lungs and then cause a series of diseases [22]. Furthermore, microplastics are described as being ingested by marine organisms and entering the food chain in this way; if there are microplastics identified in human feces, this conclusion may be proven [23,24,25].

### 2.2. Impact on Human Health

Microplastics pose potential health risks to humans, primarily through their ingestion and inhalation. The following sections summarize the key health concerns.

Oxidative Stress: Microplastics have been shown to enhance the production of ROS in biological systems, breaking the intracellular REDOX balance, which can lead to cellular damage, apoptosis, and necrosis [26]. Microplastics enhance the intestinal toxicity and are associated with ROS production, which can cause mucosal barrier injury through the North signaling pathway. Moreover, reactive oxygen species production related to the autophagy pathway promotes organoid differentiation via the Notch signaling pathway and adjusts organoid apoptosis through the OS pathway. ROS can also cause lipid peroxidation, damage cell membranes and organelles, and inhibit the activities of antioxidant enzymes, such as SOD and GPx [27]. The mechanisms underlying oxidative stress include mitochondrial dysfunction, inflammation, and direct interactions with cellular components, and they further lead to DNA damage, protein oxidation, and activation of inflammatory signaling pathways, such as NF-κB [28].

Toxicological Effects: Ways such as ingestion, inhalation, dermal contact, and biological distribution mainly caused toxicological effects [13,29]. have identified various toxicological effects associated with microplastic exposure, including inflammation, oxidative damage, and endocrine disruption [25,30,31,32] and lead to health consequences in the digestive, immune, respiratory, reproductive, and nervous systems.

Inflammation: Microplastics can trigger immune responses, leading to chronic inflammation in the affected tissues [33,34]. This inflammation can further exacerbate oxidative stress conditions. Lung epithelial BEAS-2B can be used to expound the association between pulmonary toxicity and microplastics. It has been proven that microplastics can cause cytotoxic and inflammatory effects in BEAS-2B cells through inducing ROS. They can disrupt the protective pulmonary barrier and increase lung disease risks [35].

Bioaccumulation: Microplastics may bioaccumulate in biological systems, leading to long-term exposure effects and risks to human health due to the potential leaching of harmful additives and associated contaminants [36,37]. In most cases, smaller microplastics can be ingested easily; however, bioaccumulation will be reduced if it is further reduced to a nanoscale size [38].

The content regarding the impact on human health has already been presented in Table 1 summary of the impact of microplastics on human health.

## 3. Oxidative Stress Mechanisms Induced by Microplastics

### 3.1. Understanding Oxidative Stress

Oxidative stress results from an imbalance between ROS production and cellular antioxidant defenses. ROS include various reactive molecules, such as superoxide anions (O_2_^−^), hydrogen peroxide (H_2_O_2_), and hydroxyl radicals (·OH), which are produced during normal cellular metabolism but can also arise from environmental stressors, like microplastics. When ROS levels exceed the neutralizing capability of antioxidants, oxidative stress occurs, leading to cellular injury and tissue damage by impairing normal physiological functions, releasing inflammatory mediators, and inducing inflammatory responses.

### 3.2. Molecular Mechanisms of Microplastic-Induced Oxidative Stress

Microplastics can induce oxidative stress through several intricate mechanisms.

Phagocytosis and Immune Activation: Microplastics can contact humans in many ways and cause different consequences, such as oxidative burst and temporary immunosuppression, which may disrupt immune function. Upon entering biological systems, microplastics can be recognized by immune cells, leading to phagocytosis. This process triggers an oxidative burst characterized by the rapid release of ROS and pro-inflammatory cytokines (e.g., TNF-α and IL-6) [39]. This immune response is a critical mechanism for neutralizing foreign particles; however, excessive production of ROS can lead to oxidative damage in surrounding tissues and, if sustained over time, can result in chronic inflammatory conditions that compromise overall health [40]. Temporary immunosuppression can result from reduced dendritic cell activation, increased production of anti-inflammatory cytokine IL-10, and the suppression of T-helper type 2 (Th2) responses. These mechanisms impair T-effector cell production, weakening the immune system’s ability to respond effectively to pathogens [41]. Microplastics (MPs) are engulfed by immune cells, such as macrophages and neutrophils, triggering the activation of NADPH oxidase 2 (Nox2)—a key enzyme source for oxidative burst. Microplastics (MPs) mainly activate the phagocyte NADPH oxidase (Nox2) through the TLR4-dependent pathway, generating oxidative stress and driving cellular senescence [42,43]. Nanoparticles also induce Nox2-mediated ROS through microglial cell activation, exacerbating neuronal damage [44]. Comparative studies have shown that nanoparticles/microparticles increase cardiovascular inflammatory markers through Nox2-dependent mechanisms [45]. Therapeutic inhibition of Nox2 using hyaluronic acid nanoparticles indicates the potential for reducing ROS, highlighting the central role of Nox2 in particle-induced toxicity [46,47].

Mitochondrial Dysfunction: Mitochondria are integral to energy production in cells and are a significant source of ROS during normal metabolism. Microplastics can disrupt mitochondrial function by obstructing the activity of ETC complexes, causing a leakage of electrons from complex I (NADH dehydrogenase) and III (cytochrome bc1), and generating superoxide anions [48,49,50,51]. This mitochondrial dysfunction not only results in higher ROS levels but also impairs ATP production because oxidative phosphorylation reduces ATP synthesis, leading to energy shortages within the cell. Research has shown that exposure to microplastics can lead to decreased mitochondrial membrane potential and increased mitochondrial permeability transition, which can trigger apoptosis and cell death [52,53]. Additionally, NPs may activate p66Shc, promoting mitochondrial oxidative stress [54]. In nerve cells, NPs induce mitochondrial autophagy through the AMPK/ULK1 pathway, indirectly increasing ROS [55]. Microplastic exposure can also cause anomalous opening of the mitochondrial permeability transition pore (mPTP), leading to the release of cytochrome C and the activation of the caspase cascade, eventually triggering programmed cell death. At the same time, microplastics can cause intestinal inflammation reflected in the increased level of IL-6 and TNF-α, which may eventually intensify mitochondrial oxidative damage by the way of systemic inflammatory responses [56,57]. However, it is not yet clear whether MAO or mitochondrial NOX4 is involved in this process. We believe that the surface charge of NPs may affect their interaction with the mitochondrial membrane, thereby altering the ROS production pathway [58]. However, this hypothesis requires experimental verification. Future research should combine MPs/NPs of different particle sizes and surface modifications to systematically analyze their differential effects on the source of mitochondrial ROS.

Chronic Inflammation Response: Chronic inflammation associated with microplastic exposure can perpetuate oxidative stress. Inflammatory mediators stimulate ROS production as a part of the immune response. Thus, a vicious cycle may develop, where inflammation enhances oxidative stress, and oxidative stress compounds inflammation [59]. Inflammation exacerbates oxidative stress. Inflammatory cells, like macrophages and neutrophils, can release a lot of ROS when responding to pathogens and tissue damage. Also, some inflammatory factors such as TNF-α, IL-1β, and IL-6 can further stimulate ROS production in cells and cause oxidative stress in chronic inflammatory conditions. On the other hand, ROS can activate signaling pathways directly by oxidizing proteins, such as lipids and DNA, causing an inflammatory response. The case in point is NF-κB, which regulates the expression of pro-inflammatory genes, including cytokines, chemokines, and adhesion molecules, which can exacerbate tissue damage and promote conditions like fibrosis and cancer [28,60]. Chronic oxidative stress and inflammation cause tissue damage and impair repair mechanisms. ROS and inflammatory mediators damage endothelial cells, increasing Vaso permeability and accelerating fibrosis. Cancer can be regarded as a type of chronic inflammation. In cancer, oxidative stress and inflammation drive tumor initiation, promotion, and progression, the mechanisms of which are the same as the previous description. Additionally, ROS induce DNA damage and transgenation, activate protooncogenes, or deactivate tumor suppressor genes [61].

Interaction with Cellular Components: The surface chemistry and physical properties of microplastics can facilitate their interaction with cellular membranes and other biomolecules. Studies have suggested that microplastics can integrate into cellular components, creating inflammatory and oxidative responses through cellular signaling disruptions [49,62]. These interactions can alter the membrane permeability, resulting in an ion imbalance. Additionally, the leaching of additives from microplastics, such as phthalates and bisphenol A (BPA), can further contribute to oxidative stress by interacting with various cellular pathways and disrupting hormone signaling [63]. MDA is a marker of lipid peroxidation; when microplastics cause oxidative stress, the level of MDA significantly increases, and cells exposed to microplastics undergo morphological changes, such as cell shrinkage and loss of membrane integrity, suffer more severe alterations, and have higher apoptosis rates.

Direct DNA Damage: Specific studies have shown that exposure to microplastics can result in the formation of DNA adducts and other forms of DNA damage, contributing to genetic instability [64]. DNA damage includes base oxidation, single-strand breaks, double-strand breaks, and crosslinking damage. Although cells possess DNA repair mechanisms, like base excision repair (BER) and nucleotide excision repair (NER), cellular dysfunction still exists. This damage is often mediated by reactive species generated during oxidative stress. The accumulation of DNA damage can lead to mutations, oncogenesis, cellular senescence, and genomic instability, which will increase the risk of cancer and chronic diseases. Moreover, it can affect future generations through genotoxicity [65,66,67,68]. Studies have shown that polystyrene microplastics (PS-MPs) cause plant DNA damage through direct physical damage, oxidative stress (ROS bursts and antioxidant system imbalances), and epigenetic interference. Nanoscale PS-MPs penetrated the cell, causing DNA strand breaks, ROS excessive accumulation of attacking bases, and inhibiting repair enzyme activity. PS-MPs can also be used as a contaminant carrier to aggravate damage. The transgenerational genetic effects still need to be further studied [69].

Endoplasmic Reticulum (ER) stress: Microplastic exposure may induce ER stress, leading to an unfolded protein response (UPR). UPR activation can enhance ROS generation by disrupting redox homeostasis while attempting to restore normal cellular function [70]. Persistent ER stress may also trigger apoptotic pathways, further exacerbating oxidative damage. This is particularly crucial because ER stress can activate several cell survival or death pathways related to inflammation and oxidative stress, contributing to tissue damage [62,71,72]. Studies showed that food pollutants are potentially harmful in neurodegenerative diseases, which promote the transcription of pro-apoptotic genes by upregulating the expression of CHOP. The JNK pathway was activated to induce apoptosis. The disruption of calcium homeostasis leads to ER dysfunction. In addition, oxidative stress induced by pollutants increases the production of reactive oxygen species (ROS), which further damages ER membranes and protein folding machinery. The inflammatory response is also activated, releasing inflammatory factors through the NF-κB pathway, exacerbating ER stress and neuronal apoptosis. This underscores the importance of reducing pollutant exposure to protect neuronal health [73].

MicroRNA Regulation: Emerging evidence indicates that microplastics can modulate the expression of microRNAs (miRNAs) related to oxidative stress and inflammatory responses. Dysregulated miRNAs can contribute to cellular responses to oxidative stress and affect the pathways involved in cell survival and apoptosis [74]. Such epigenetic alterations can lead to long-term changes in cellular function and promote carcinogenesis [75,76]. Studies found that Exosomal miRNAs may indirectly mediate energy metabolism disorders in intestinal epithelial cells and reduce the expression of tight junction proteins (such as ZO-1 and occludin) by regulating the expression of genes related to the AMPKα pathway, thereby destroying the integrity of the intestinal barrier. Some miRNAs may target AMPKα or its upstream regulators, leading to pathway inhibition and further aggravating intestinal barrier dysfunction [77].

**Figure 1:**
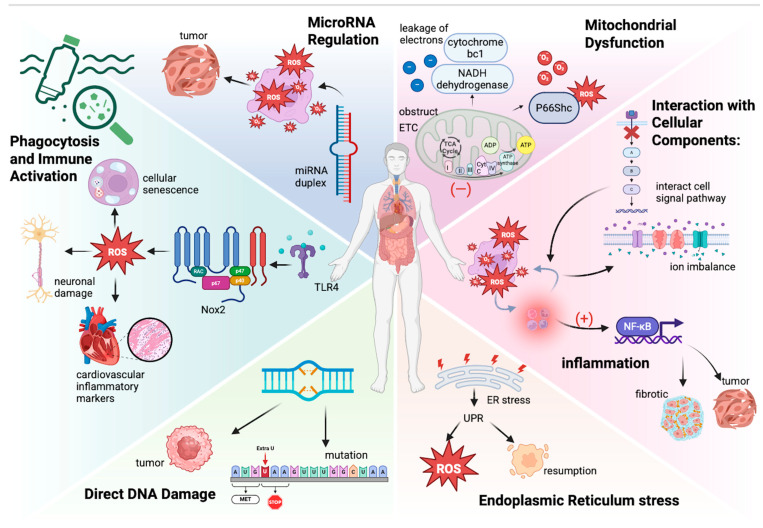
Mechanisms of microplastics induce reactive oxygen species [39,40,41,48,49,50,51,52,53,54,55,56,57,58,59,60,61,62,63,64,65,66,67,68,69,70,71,72,73,74,75,76,77].

The content regarding the molecular mechanisms of oxidative stress induced by microplastics has been introduced in Figure 1 Mechanisms of microplastics induce reactive oxygen species [39,40,41,48,49,50,51,52,53,54,55,56,57,58,59,60,61,62,63,64,65,66,67,68,69,70,71,72,73,74,75,76,77].

### 3.3. Evidence of Oxidative Stress Induction

Numerous studies have demonstrated that microplastics induce oxidative stress in various biological models.

In Vitro Evidence: For instance, a study involving human lung epithelial cells exposed to microplastics reported significant increases in ROS levels and markers of oxidative damage, including lipid peroxidation and protein carbonylation [78,79]. The ROS increase showed dose- and time-dependent patterns, which means that the ROS level rose when exposed to higher microplastic concentrations and longer exposure durations. Next, lipid peroxidation levels also increased, which indicates that cell membranes suffered oxidative damage because of excessive ROS generation. Additionally, protein carbonylation levels increased significantly, which shows that the protein structures have changed, which will influence cellular function, stability, and cell viability. Moreover, microplastics can also cause cell damage by apoptosis, physical damage, chemical toxicity, inflammatory reaction, and so on [80]. Such biochemical alterations indicate cellular injury, suggesting that microplastics can inflict a substantial oxidative burden [81].

In Vivo Evidence: Animal studies have consistently highlighted that exposure to microplastics elevates oxidative stress markers in multiple organs, including the lungs, liver, and kidneys. Elevated levels of malondialdehyde (MDA) and decreased glutathione (GSH) levels were observed, indicating oxidative damage and the depletion of antioxidant defenses [82,83,84]. Moreover, histopathological examinations of tissues revealed lesions and inflammation as a result of sustained oxidative stress, reinforcing the link between microplastic exposure and detrimental health impacts. In vivo animal studies have shown that exposure to microplastics can cause damage to lung epithelial cells and pulmonary fibrosis. This damage occurs through the production of inflammatory mediators (such as TNF-α and macrophage-derived factors). Long-term exposure to microplastics may lead to chronic lung diseases [85]. Recent studies using zebrafish models have shown that polyethylene nanoplastics can cause oxidative stress, leading to developmental and physical damage. Exposure to PS NP triggers a surge in reactive oxygen species, reduces antioxidant defenses, such as the activity of SOD and CAT and the decrease in GSH levels, and increases lipid peroxidation. Mitochondrial dysfunction is an important consequence, characterized by a loss of membranes, ATP, and structural damage. In the transfer with arsenic (As), PS-NPs show a synergy that allows for greater propulsion and strengthening of mitochondria. In addition, neurotransmitters are weakened due to oxidative stress and precipitation of dopamine and serotonin levels. These results highlight that drivers are at the heart of nanoplastic poisoning, which links the oxidation of aquatic organisms to greater physical damage [86,87]. The harm of microplastics (MPs) to human health has been verified in multiple dimensions: First, the study detected PET, PS, and other plastic particles in human blood and confirmed that they can be distributed throughout the body through the circulatory system [88]. Second, clinical evidence shows that MPs, such as polyethylene and polyvinyl chloride, are enriched in atherosclerotic plaques in patients with acute coronary syndrome, accompanied by increased vascular complexity and cardiovascular risk [89]. In addition, experimental studies have shown that MPs can induce oxidative stress, inflammatory response, and endothelial dysfunction, thereby promoting cardiovascular disease and metabolic disorders [25]. These findings suggest that MPs not only accumulate in the human body but also may harm health through pro-inflammatory and pro-atherogenic mechanisms, and it is urgent to establish systematic risk assessment and prevention and control strategies.

Additionally, studies have used biomarkers of oxidative stress, such as protein carbonyls and oxidative DNA damage (8-OHdG), to assess the extent of damage caused by microplastics. The results showed significant increases in these biomarkers correlating with exposure doses and durations, thus providing compelling evidence for the toxicological effects of microplastics on cellular health [90,91,92].

## 4. Antioxidant Interventions: Mechanisms and Efficacy

### 4.1. Types of Antioxidants

Antioxidants can be classified into enzymatic and nonenzymatic antioxidants.

Enzymatic Antioxidants: This category includes superoxide dismutase (SOD), catalase (CAT), and glutathione peroxidase (GPx). These enzymes are produced endogenously and play critical roles in neutralizing ROS during cellular metabolism [93,94]. Their activity is essential for maintaining the cellular redox balance and preventing oxidative damage [14,95,96]. Here, we use SOD as an example. The antioxidant mechanism of superoxide dismutase catalyzes the conversion of superoxide radicals (O_2_^−^) into hydrogen peroxide (H_2_O_2_) and oxygen O_2_) and plays a key role in maintaining redox balance to prevent the over-accumulation of ROS. ROS are not only toxic molecules but are important signaling molecules, which participate in the possesses of cell proliferation, differentiation, and apoptosis. SOD influences redox signaling pathways such as NF-κB, MAPK, and PI3K/Akt by adjusting the level of ROS. There is a dynamic equilibrium in the generation and metabolism of ROS by the effect of SOD [97].

Non-Enzymatic Antioxidants: These antioxidants include compounds, such as vitamins C and E, carotenoids, flavonoids, and polyphenols, which can be obtained from dietary sources [98]. They act as free radical scavengers and can effectively mitigate oxidative stress [99,100]. Common sources that we use to intake carotenoids include carrots, tomatoes, spinach, citrus fruits, and egg yolks. They play an important role in protecting the eye, skin, and cardiovascular health and preventing cancer [101]. The relevant information about the types of antioxidants has been organized in Table 2 Summary of the classification and mechanism of action of various antioxidants.

### 4.2. Mechanisms of Action

Antioxidants can combat oxidative stress through multiple mechanisms.

Direct Scavenging of ROS: Many antioxidants can directly neutralize ROS, thereby reducing their potential for cellular damage. For example, vitamin E can effectively scavenge lipid peroxyl radicals in cell membranes, thereby protecting cellular integrity [102]. Vitamin C, which is water soluble, can scavenge ROS in the cytosol and extracellular fluid, providing a joint defense mechanism alongside lipid-soluble antioxidants [103,104]. Carotenoids fulfill the role of antioxidants by directly scavenging free radicals (singlet oxygen and superoxide anions) and quenching singlet oxygen. They can work synergistically with vitamin C and vitamin E as well. Carotenoids play an important role in certain cancers, such as lung and prostate cancer, by reducing DNA oxidative damage [101].

Regeneration of Other Antioxidants: Some antioxidants can regenerate others, thereby enhancing the overall antioxidant capacity of the cell. This synergistic interaction is essential for maintaining the cellular redox balance, as oxidized antioxidants can be restored to their active forms. For instance, vitamin C can scavenge free radicals and neutralize them by donating electrons, vitamin E can protect cell membranes from oxidative damage by interrupting lipid peroxidation chain reactions, and vitamin C can regenerate oxidized vitamin E, prolonging its protective effect by securing polyunsaturated fatty acids (PUFAs) from oxidation, decreasing the production of reactive oxygen species, regulating signal transduction, and strengthening the overall antioxidant defense system [105,106]. Vitamin C and vitamin E have functions like delaying Alzheimer’s and Parkinson’s by protecting neurons from oxidative damage and controlling the occurrence of tumors by reducing DNA oxidative damage, and they have the same mechanisms in diabetes, cataracts, and some age-related diseases [107].

Modulation of Antioxidant Enzyme Expression: Antioxidants can upregulate antioxidant defense systems by modulating signaling pathways, such as the Nrf2 pathway [108,109]. The activation of Nrf2 leads to the translocation of the Nrf2 protein to the nucleus, where it binds to antioxidant response elements (AREs) in the promoter regions of antioxidant genes [110], resulting in an increased expression of endogenous antioxidants, such as SOD, CAT, and GPx [111,112]. This transcriptional activation results in enhanced cellular resistance to oxidative stress and decreases ROS [113]. In contrast, the expression of glutathione synthesis enzymes (e.g., GCL) increases, which can improve the level of intracellular glutathione. Many natural compounds, such as curcumin and sulforaphane (SFN), exhibit antioxidant and anticancer effects by activating Nrf2 [114].

Enhancing Mitochondrial Function: Some antioxidants, such as nobiletin, have shown the ability to directly enhance mitochondrial function [115,116]. Nobiletin sensibly improved the expression of mitochondrial biogenesis-related genes, such as PGC-1α, NRF1, and TFAM, and raised mtDNA content. Nobiletin can ameliorate the mitochondrial function and increase mitochondrial membrane potential (MMP) and ATP production [117]. Kaempferol upregulates the expression of downstream antioxidant genes, such as HO-1 and GCLC, by activating the Nrf2 pathway, significantly enhancing the antioxidant defense ability of hepatocytes, effectively inhibiting paracetamen-induced lipid peroxidation and iron death, and ultimately preventing liver function injury (manifested by reduced ALT and AST levels). This finding provides a new way to prevent and treat drug-induced liver injury [118]. Mitochondria-targeted antioxidants, such as MitoQ, specifically target mitochondria to directly mitigate oxidative stress at its source [119].

Attenuating Inflammatory Responses: By inhibiting pro-inflammatory signaling pathways or decreasing cytokine release, antioxidants can help reduce the inflammatory component of oxidative stress [120]. Astaxanthin (AST) inhibits the key signaling pathways of anti-inflammatory factors, such as NF-κB and MAPK, and reduces the production of TNF-α, IL-6, and IL-1β to further reduce inflammatory responses, eliminate ROS, and inhibit oxidative stress [121]. This action may further diminish ROS production, creating a positive feedback loop that enhances cellular health. These mechanisms can help to improve endothelial function, relieve insulin resistance, and protect neurons.

Induction of Cell Survival Pathways: Some antioxidants can activate cell survival pathways that promote cell repair and proliferation in the face of stress [122]. For instance, certain flavonoids have demonstrated the ability to activate the PI3K/Akt pathway, enhance cell survival, and reduce apoptotic signals [123,124,125]. In a study, the researchers used a rat ischemia/reperfusion(I/R) model and found that quercetin alleviated cerebral I/R injury by improving neurological deficits. One of the molecular mechanisms is quercetin, which activates the PI3K/Akt signaling pathway and promotes the expression of M2 polarization-related genes to mitigate inflammatory responses and enhance neuroprotection [126]. This protective mechanism may be vital in preventing cell death and maintaining tissue integrity after exposure to damaging agents, such as microplastics.

Enhancement of Detoxification Mechanisms: Antioxidants may also enhance cellular detoxification systems, which are crucial in processing and expelling harmful xenobiotics, including plastic-derived chemicals [127]. This process is essential for mitigating the effects of prolonged exposure. An increase in phase II detoxifying enzymes, such as glutathione S-transferases (GSTs), has been noted in cells treated with certain antioxidants, indicating their role in supporting detoxification pathways [128]. GSTs play an important role in cancer chemoprotection by decreasing the level of ROS and detoxifying carcinogens. A higher level of GST is highly associated with a lower risk of lung and liver cancer, which are populations exposed to environmental carcinogens, particularly. GSTs can also regulate apoptosis and cell proliferation signaling pathways, which can participate in the prevention of cancer indirectly [129].

MicroRNA Regulation: In the context of microplastic exposure, certain antioxidants have been shown to modulate the expression of miRNAs that play a key role in regulating oxidative stress responses in cells [130]. By influencing miRNA expression, antioxidants may help restore balance to the cellular oxidative state [131]. Here, we use type 2 diabetes (T2D) as an example. Certain miRNAs (miR-146a, miR-34a, miR-200c, etc.) regulate intracellular redox balance by targeting antioxidant enzymes (e.g., SOD, CAT) or oxidative stress-related signaling pathways (e.g., Nrf2/ARE, NF-κB). On the other hand, oxidative stress can influence miRNA expression profiles by altering miRNA biogenesis or degradation processes. Moreover, miRNAs can participate in the chronic inflammatory response by regulating the expression of inflammatory factors (e.g., TNF-α and IL-6). The abnormal expression of these certain miRNAs can be found in T2D patients and may serve as potential biomarkers [132].

**Figure 2:**
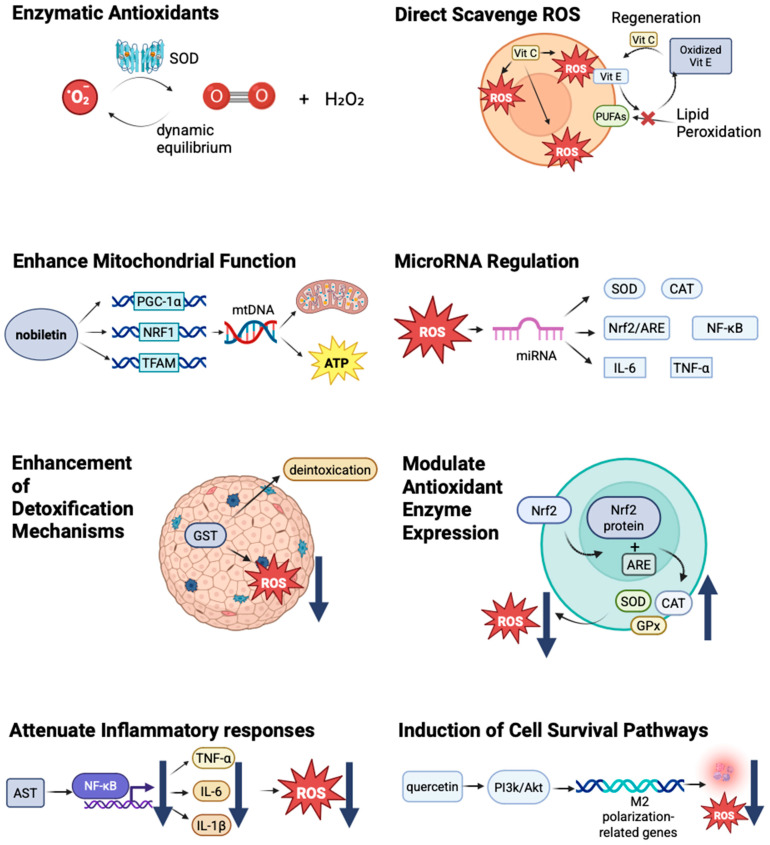
Different mechanisms by which antioxidants mitigate oxidative damage [102,103,104,105,106,107,108,109,110,111,112,113,114,115,116,117,118,119,120,121,122,123,124,125,126,127,128,129,130,131,132]. ↑ indicates that a certain substance is upregulated or increased. ↓ indicates that a certain substance is downregulated or reduced.

The mechanism by which antioxidants counteract oxidative stress has been illustrated in Figure 2 Different mechanisms by which antioxidants mitigate oxidative damage [102,103,104,105,106,107,108,109,110,111,112,113,114,115,116,117,118,119,120,121,122,123,124,125,126,127,128,129,130,131,132].

### 4.3. Evidence Supporting Antioxidant Efficacy

#### 4.3.1. In Vitro Studies

Several in vitro studies have investigated the protective effects of antioxidants against microplastic-induced oxidative stress.

Vitamin C: Co-treatment with vitamin C significantly reduces oxidative stress markers in human lung fibroblasts exposed to microplastics (e.g., SA-β-gal activity, p16, and p21 expression) [133]. The study demonstrated that vitamin C lowered ROS levels and improved cell proliferation and function, highlighting its role as a potent antioxidant in preventing microplastic-induced injuries. Mechanistically, vitamin C directly scavenges cytosolic ROS (e.g., hydroxyl radicals) and regenerates oxidized vitamin E, synergistically protecting lipid membranes from peroxidation [102,103]. Mechanistic studies showed that vitamin V relieved PM-induced oxidative stress by directly scavenging ROS and prevented cellular senescence by inhibiting the p16/p21 signaling pathway [134]. Studies have found that dietary vitamin C may inhibit abdominal aortic calcification (AAC) by reducing oxidative stress through antioxidant effects, and its high intake is associated with a lower risk of AAC [135].

Curcumin: Research has shown that curcumin, a natural polyphenolic compound with potent antioxidant properties, can mitigate oxidative damage in cells exposed to microplastics by scavenging ROS and enhancing the expression of antioxidant enzymes [136]. Curcumin activates the Nrf2/ARE pathway, upregulating HO-1 and GST to enhance cellular detoxification capacity [111,114]. Curcumin has been found to activate Nrf2, thereby increasing cellular resilience to oxidative stress [111]. In mechanistic studies, researchers used the RAW 264.7 cell model and treated the cells with H_2_O_2_. The experimental results showed that curcumin improved the viability of cells, alleviated oxidative stress, and improved the activity of antioxidative enzymes. Additionally, curcumin activated the Nrf2-Keap1 signaling pathway and promoted the nuclear transcription of Nrf2 and the upregulation of downstream target genes (e.g., HO-1), which can further enhance the cellular antioxidant defense capacity.

Quercetin: A study indicated that quercetin (QSN) treatment significantly decreases oxidative stress in human intestinal cells exposed to microplastics [137], providing a mechanism for its protective effects against inflammation and cellular damage. Quercetin activates the PI3K/Akt pathway to promote cell survival and inhibits NF-κB-mediated inflammation, reducing TNF-α and IL-6 release [126,138]. QSNs exert antioxidant and anti-inflammatory effects by scavenging ROS and inhibiting the NF-κB and MAPK signaling pathways, which can significantly reduce the expression of anti-inflammatory factors (e.g., TNF-α and IL-6). The nanostructural properties of QSNs can allow them to target intestinal tissues more efficiently, and QSNs have prominent anticancer activities, thereby regulating the cell cycle, inducing apoptosis, and inhibiting tumor cell proliferation [138].

Resveratrol: Resveratrol has also been studied for its protective effects, particularly its ability to decrease ROS levels and increase the activity of antioxidant enzymes, in various cell models exposed to microplastics [139,140]. Resveratrol enhances mitochondrial biogenesis via PGC-1α/NRF1/TFAM signaling, improving ATP production and reducing mtROS [117]. Its role in upregulating heme oxygenase-1 (HO-1), which has antioxidant properties, concurs with its protective effects against oxidative stress. Simultaneously, resveratrol improves mitochondrial function, reducing mitochondrial ROS production and further lowering oxidative stress levels. At the cellular level, resveratrol maintains cellular homeostasis by protecting cell membranes, organelles, and DNA from oxidative stress.

Melatonin: It has been proven that MT can enhance plant tolerance to abiotic stresses through multiple mechanisms. Melatonin (MT) scavenges mitochondrial ROS (mtROS) and upregulates SOD/CAT activity while modulating Ca^2+^ signaling and stress-responsive genes (e.g., DREB/WRKY) [141,142]. As a potent antioxidant, MT scavenges reactive oxygen species (ROS) and upregulates antioxidant enzyme activity to maintain REDOX homeostasis. It regulates Ca^2+^ signaling, the MAPK cascade, and plant hormone pathways, activates stress resistance genes, such as DREB and WRKY, and optimizes the stress response. MT also enhances stress memory and long-term stress resistance by affecting DNA methylation, histone modifications, and non-coding RNA expression [142]. In addition, MTS synergistically enhances plant defense against pathogens and induces cross-resistance. Exogenous MT treatment provides a new strategy for cultivating stress-resistant crops [131,141,143]. Using a rat model, it was found that polyethylene microplastics (PE-MPs) caused dose-dependent damage to the adrenal cortex in rats, which was manifested by decreased cortisol levels and histopathological changes. Melatonin (5/10 mg/kg) alleviated the damage in a dose-dependent manner, and the effect of 10 mg/kg was more significant. The study suggests that melatonin has a dose-dependent protective effect on endocrine disturbance induced by PE-MPs [144]. In terms of respiratory exposure, respirable textile microplastic fibers have been shown to impair airway epithelial cell differentiation, disrupt respiratory barrier function, and increase the risk of respiratory diseases [145]. However, the environmentally relevant concentrations of microplastics exposed through the digestive tract can cause bile acid metabolism disorder and cholestasis by disturbing intestinal flora and activating the gut–liver axis signaling pathway, suggesting indirect toxicity to the liver [146].

Combination Studies: Recent studies have explored the synergistic effects of multiple antioxidants. Vitamin E and selenium co-administration enhances GPx activity and reduces lipid peroxidation, offering superior protection against MP-induced mitochondrial dysfunction. The preliminary results suggest that combinations of antioxidants, such as vitamin E and selenium, can provide enhanced cytoprotective benefits in cellular models challenged with microplastics [147].

In conclusion, these findings indicate that microplastics can disrupt crucial physiological functions, whether through direct respiratory contact or indirect intestinal contact. Their health risks are systemic, affecting multiple organs, and they emphasize the necessity of assessing the cumulative effects of long-term low-dose exposure.

#### 4.3.2. In Vivo Studies

In vivo studies have further supported the protective role of antioxidants.

Glutathione: A study demonstrated that glutathione supplementation in rats exposed to microplastics significantly decreased markers of oxidative stress in the liver [148], while Gly combined with N-acetylcysteine (NAC), improving mitochondrial function, as evidenced by the restoration of mitochondrial membrane potential and increased ATP levels, indicating the effectiveness of this endogenous antioxidant in combating microplastic-induced toxicity. Otherwise, GlyNAC promoted mitophagy, reducing the accumulation of abnormal mitochondria and regulating nutrient sensing pathways (e.g., mTOR and AMPK), which improved cellular metabolic status. GlyNAC also reduced genomic damage, as indicated by decreased DNA breaks [149]. Increased levels of GSH effectively neutralized ROS, showcasing its critical protective role.

Resveratrol: Dietary resveratrol has been shown to alleviate oxidative stress and inflammation in mice exposed to microplastics, underscoring the potential of natural dietary antioxidants to protect against environmental toxins [120]. The results indicated that resveratrol significantly decreased the levels of ROS and MDA, alleviated oxidative stress, suppressed the expression of neuroinflammatory factors, and mitigated neuroinflammation. Mechanism studies showed that resveratrol enhanced cellular antioxidant capacity by activating the SIRT1 signaling pathway and reducing the release of pro-inflammatory factors through SIRT1-mediated NF-κB inhibition, as evidenced by reduced p65 phosphorylation. It also activated the Nrf2 signaling pathway, upregulated HO-1 expression, and decreased inflammatory mediator levels while enhancing antioxidant enzyme activities [150,151].

Astaxanthin: Research on astaxanthin, a carotenoid with powerful antioxidant properties, demonstrated its ability to protect against oxidative stress induced by microplastics in a mouse model [152]. In mechanism studies, astaxanthin not only scavenged free radicals but also improved mitochondrial function and decreased inflammatory responses, suggesting its potential as a therapeutic agent [153]. Additionally, astaxanthin activated the SIRT1 signaling pathway, inhibited NF-κB activity and Ac-p65 expression, and improved histopathological changes and cellular function [154].

Beta-Carotene: Studies indicate that beta-carotene supplementation mitigates oxidative stress induced by microplastics in rat models by enhancing the activity of various antioxidant enzymes (e.g., SOD, CAT, GPx) and exerts anticancer effects by inducing apoptosis and inhibiting the cell cycle, highlighting its role in bolstering the body’s defense mechanisms against microplastic exposure [155,156].

Additionally, combination studies investigating the effects of multiple antioxidants have reported enhanced protective outcomes compared with single-agent treatments, suggesting potential synergistic effects in combating microplastic-induced oxidative stress.

The differences in sources, bioavailability, dose effects, and limitations of various antioxidants have been clearly stated in Table 3 Conclusions of the differences of sources, bioavailability, dose–effects, and limitations of different antioxidants.

### 4.4. Mechanistic Insights

The mechanisms underlying the protective effects of antioxidants in the context of microplastic exposure are as follows.

Reduction in Inflammatory Response: Through the suppression of certain signaling pathways, antioxidants may lower the production of inflammation-related molecules while reducing oxidative stress linked to inflammatory processes [170]. That means that this approach helps manage cellular responses that could lead to tissue damage. For example, natural compounds, like curcumin from turmeric and quercetin, found in apples have been observed to hinder the activation of these pathways, thereby decreasing the release of pro-inflammatory substances. To put it simply, these substances work by calming excessive immune reactions in the body [171]. Plant-based components, such as lignans—including subinosin and enterolactone types—operate through multiple mechanisms. They reduce levels of inflammatory markers, like tumor necrosis factor-alpha and various interleukins, as well as nitric oxide production. This occurs through interference with cellular communication pathways while simultaneously enhancing antioxidant defense systems. In other words, such compounds address inflammation both directly and indirectly by combining pathway inhibition with the activation of protective responses. The combined effects result in comprehensive inflammation management through different biological routes [172]. Lentinus edodes reduced LPS-induced inflammatory response and apoptosis by activating the Nrf2 pathway, enhancing the expression of HO-1 and SOD and inhibiting the activity of NF-κB [173]. These studies reveal the potential application of lignans and lentinan in chronic inflammatory diseases and infection-related inflammation [174,175].

Mitigation of Mitochondrial Dysfunction: Certain antioxidants can help restore mitochondrial function, thereby reducing mitochondrial ROS production and improving the overall cellular health [176]. This restoration is critical for maintaining energy metabolism and minimizing oxidative stress. For instance, coenzyme Q10 has been shown to restore mitochondrial function and reduce oxidative stress in various models, given the energy demands of cells facing toxic insults from microplastics [115].

Enhancement of Endogenous Antioxidant Capacity: Antioxidants that activate the Nrf2 pathway help boost the synthesis of endogenous antioxidant enzymes, providing enhanced protection against oxidative stress derived from microplastic exposure. Studies have documented that compounds, such as sulforaphane, can significantly increase the levels of antioxidant enzymes, thus fortifying a cell’s defense mechanisms [112].

## 5. Advantages of Antioxidant Interventions

### 5.1. Multifaceted Protection

The ability of antioxidants to target multiple pathways involved in oxidative stress and inflammation offers a multifaceted approach to counteract the adverse effects of microplastics by neutralizing ROS, enhancing antioxidant enzyme activity, such as SOD, CAT, and GPx, and inhibiting pro-inflammatory signaling, reducing cytokine release, such as TNF-α and IL-6. Additionally, antioxidants protect mitochondrial function and support cellular recovery. Broad-spectrum protection is vital for maintaining cellular health and preventing disease progression.

### 5.2. Nutritional Accessibility

Many antioxidants, particularly dietary antioxidants, are readily available in fruits, vegetables, and other food products. This accessibility makes them attractive candidates for nutritional interventions aimed at reducing the health risks associated with microplastics. For instance, bitter melon saponins are natural active ingredients extracted from bitter melons, with antioxidant and anti-aging properties. Sirtuin 2.1 (SIR 2.1) and Helix–Loop–Helix 30 (HLH-30) are the key regulatory factors in the IIS pathway, which are closely related to oxidative stress and aging. Future research should further investigate the antioxidant and anti-aging effects and mechanisms and the synergistic effects, evaluating efficiency and safety in humans [177].

### 5.3. Potential for Public Health Strategies

Understanding the protective role of antioxidants provides valuable insights into the development of public health strategies focused on mitigating microplastic exposure and its health impacts. Dietary recommendations emphasizing antioxidant-rich foods, such as fruits, vegetables, nuts, seeds, and whole grains, which are rich in vitamins C and E, beta-carotene, and polyphenols, can be a proactive approach to enhancing health resilience in populations at risk, and targeted intervention measures can be developed for populations with higher exposure risk. Developing policies aimed at reducing plastic pollution and improving waste management can address the health risks caused by microplastic exposure at the source. Interventions can be stratified for the general population. For example, for regions, there are different interventions for people in high-risk areas of microplastic pollution and people at low risk of microplastic pollution [178]; for individuals, the risk of microplastic ingestion may vary greatly in some jobs. Therefore, individualized antioxidant supplementation programs should be designed for individuals with a high risk of microplastic exposure [179,180]. In addition, we can set up dietary guidelines to help different individuals find their own antioxidant regimen [181]. Studies suggest that personalized dietary recommendations, such as Caloric Restriction, may activate the AMPK/SIRT1 pathway and enhance autophagy and antioxidant capacity, provided that the risk of malnutrition is avoided and the Mediterranean diet pattern is avoided. Consumption of a diet rich in olive oil, fish, nuts, and fruits and vegetables, which provide polyphenols, antioxidants, and anti-inflammatory components, may delay ALS progression. However, it needs to be carefully selected according to its own situation and the degree of microplastics exposure [182].

### 5.4. Low Toxicity and Side Effects

Compared to synthetic pharmacological agents, many dietary antioxidants such as vitamins C and E, beta-carotene, and polyphenols have a favorable safety profile and are associated with minimal side effects, making them suitable for long-term use and broader population applicability; this favorable safety profile is supported by extensive research, which has demonstrated that these natural antioxidants cause significant harm when rarely consumed in recommended amounts [183,184]. This low toxicity is important when considering widespread public health interventions that are particularly suitable for vulnerable populations, such as children, pregnant women, and the elderly [185]. In summary, this approach not only addresses immediate health concerns but also promotes long-term well-being.

### 5.5. Cost Effectiveness

Many antioxidants, particularly those derived from natural sources, are relatively inexpensive. They can be incorporated into daily diets without a significant financial burden, making them a practical choice for populations in various socioeconomic circumstances. For instance, locally grown produce and seasonal fruits often provide a cost-effective way to boost antioxidant intake, making them a viable choice for low-income populations. Additionally, they can lower the pressure on healthcare systems with an economic strain. In this study, the antioxidant activity of citrus peel powder was significantly improved using a two-stage nutritional optimization strategy combined with Aspergillus Niger solid-state fermentation, which not only provided a way for high-value utilization of citrus processing waste but also reduced environmental pollution. This green bioprocessing technology has a low cost and high efficiency and is expected to replace synthetic additives as natural antioxidants in food and healthcare products, with significant economic benefits and ecological sustainability [186].

## 6. Challenges and Limitations of Antioxidant Interventions

### 6.1. Variability in Efficacy

Antioxidant efficacy can vary widely based on several factors, including the specific type of antioxidant, dosage, and duration of exposure. For example, vitamins C and E show a strong ability to eliminate free radicals in vitro but show circumscribed effectiveness in vivo due to complex interactions within the human body. This variability complicates the clinical applications and standardization for use in public health settings [187,188,189]. Furthermore, it is difficult to develop general medical guidelines owing to a lack of consensus regarding the optimal dosage and ratio.

### 6.2. Bioavailability Concerns

Bioavailability of dietary antioxidants can significantly influence their effectiveness. Factors such as metabolism, absorption, and distribution play key roles in determining the actual amounts of antioxidants reaching target tissues. For example, polyphenols in fruits and vegetables are rapidly metabolized in the liver and intestines, so their bioavailability is difficult to control. Individual differences in genetics, gut microbiota, and overall health status further contribute to variability in response to antioxidants; therefore, variability in individual responses to dietary interventions may also impact outcomes [190].

### 6.3. Lack of Standardization in Research Protocols

Standardized research protocols are required to evaluate the effectiveness of antioxidants in various contexts. Current studies often use different methodologies, making it challenging to compare results and draw broader conclusions about antioxidant efficacy across diverse settings [178,179,180,181,182,191,192].

### 6.4. Long-Term Effects Require Further Investigation

Although many studies have demonstrated the protective effects of antioxidants against microplastic-induced oxidative stress, most have been short-term or limited in scope. Comprehensive long-term studies are essential to understand the chronic implications of antioxidant supplementation on health outcomes due to prolonged exposure to microplastics [193,194].

### 6.5. Complexity of Mixtures

Microplastics often contain a complex mixture of additives and absorbed environmental pollutants, which can complicate the interpretation of their antioxidant efficacy [195]. The interactions among antioxidants, microplastics, and other contaminants require careful investigation to understand the full scope of their effects.

### 6.6. Lack of Vitro and Vivo Experiments

In the process of searching the literature, most studies focus on the mechanism of cytology, while very few studies perform animal and human experiments. The reason may be that animal experiments have great individual differences, are not easy to obtain experimental results, and require longer experimental times and higher costs. But, as time goes on, there will be more research on this subject for experimentalists to consider.

### 6.7. Antioxidant Toxicity

Antioxidant supplements exhibit contradictory effects at high doses. Long-term high-dose intake may trigger pro-oxidative effects and disrupt the redox homeostasis, especially for specific groups, such as smokers or cancer patients [196,197]. For instance, beta-carotene supplementation amplifies ROS and increases the risk of lung cancer in smokers [198,199]. Moreover, antioxidants may interfere with the efficacy of chemotherapy/radiotherapy by neutralizing tumor-destroying ROS [200,201]. These findings emphasize the importance of dose control and population-specific risk assessment.

### 6.8. Antioxidant Contraindications

At high doses or under specific physiological conditions, antioxidants may exhibit pro-oxidative effects [202]. Patients receiving anticoagulant therapy should avoid vitamin E supplements, as they increase the risk of bleeding [203]. The combination of N-acetylcysteine (NAC) and nitroglycerin may cause severe hypotension [204]. These studies confirm that the contraindications of antioxidants depend on the dose, the patient’s comorbidities, and drug interactions. Before clinical application, individualized risk assessment is required for such medications.

### 6.9. Lack of Human Exposure Research

At present, there is still controversy over whether the internal doses of microplastics (MPs) and antioxidants are environmentally relevant. Animal studies have shown the toxicity of MPs (such as liver fibrosis induced by a dose of 10 mg/kg/day in mice [205]), but the actual human exposure is much lower (about 0.1–5 μg/kg/day through diet [206]). Most in vivo experiments on antioxidants use super-physiological doses (such as 500 mg/kg curcumin in rats [207]), far exceeding dietary intake (usually < 10 mg/kg), which casts doubt on the extrapolation of research conclusions.

New evidence suggests that low doses of MPs may still disrupt human redox balance (such as placental transfer phenomena [26]), but nutritional doses of antioxidants (such as 200 mg/day vitamin C [208]) have limited inhibitory effects on MP-induced oxidative damage in clinical practice [209]. Although some studies have proposed synergistic schemes (such as quercetin + selenium [210]), their environmental relevance needs further verification.

Key issues include (1) the dose–effect threshold of MPs on human biomarkers (such as 8-OHdG [211]); (2) the long-term safety of high-dose antioxidants [207]; and (3) the interaction with MP additives (such as phthalates) [212]. Future research should prioritize the use of environmentally relevant exposure models [213].

### 6.10. Lack of Relevant Human Epidemiological Studies

At present, there is still limited human epidemiological evidence regarding whether the intake of antioxidants can alleviate the effects of microplastic (MP) exposure. A 2025 biological monitoring study found that higher plasma vitamin C levels in the MP-exposed population were associated with a decrease in urinary 8-OHdG (an oxidative stress marker), but the causal relationship is still unclear [214]. On the contrary, a 2024 cohort study showed that dietary polyphenols had no significant interaction with MPs on inflammatory markers (CRP/IL-6) [215]. It is notable that both studies pointed out confounding factors: chemical additives in MPs (such as phthalates) may dominate the toxicity [212], while high-dose antioxidant supplements may instead have a pro-oxidative effect [216]. New data indicate that a diet rich in selenium may alleviate mitochondrial dysfunction induced by MPs [210], but larger-scale cohort studies are needed for verification. Key issues include the following: (1) longitudinal studies are needed to clarify the temporal relationship; (2) standardize the biological markers of MP exposure; and (3) stratify analysis by the source of antioxidants (food vs. supplements) [217]. The current evidence is insufficient to confirm the protective effect, and dietary recommendations should be made with caution.

## 7. Future Directions

### 7.1. Understanding Molecular Pathways

Further research is necessary to elucidate the molecular pathways involved in microplastic-induced oxidative stress and the role of antioxidants in modulating these pathways. Identifying key signaling mechanisms may lead to targeted antioxidant therapies for mitigating specific health risks associated with microplastics [218]. The synergistic mechanism of MPs and pollutants during bioaccumulation and the specific molecular pathway by which PS-MPs destroy tight junction proteins (such as ZO-1 and occludin) remain unclear [219,220]. The interaction mechanism of the various links in the lysosome/ROS/iron death pathway has not been fully elucidated [221].

### 7.2. Development of Novel Antioxidant Formulations

Exploring new antioxidant combinations and delivery methods could potentially improve effectiveness through what we might call synergistic interactions between components. That means that future research efforts should focus on creating and testing blended antioxidant formulas that may boost protective impacts against toxicity from microplastic exposure. In simpler terms, mixing different antioxidants might work better than using single ingredients alone.

In recent years, tiny drug delivery systems have been finding applications across various fields, including cancer treatment. The approach of delivering natural bioactive compounds alongside chemotherapy drugs offers a promising way to address issues like side effects and treatment resistance seen in traditional approaches. For instance, researchers have explored using liposome carriers or nanoparticles made from polymers to simultaneously transport paclitaxel with curcumin. This combination has shown potential to block certain proteins that push drugs out of tumor cells, thereby improving how well chemotherapy agents can destroy cancerous growths [222]. In the area of delivery methods, the “nano-in-nano” approach involving electrospun fibers combined with nanoparticles achieves two main benefits: combining sustained local drug release with improved delivery across biological barriers through embedding medication-carrying biodegradable particles into polymer-based fiber materials. Meanwhile, NDDS has seen important progress in genetic treatment applications by developing delivery methods for specific genetic material. For instance, these systems might use either cationic liposomes or PAMAM dendrimers modified with folic acid, which helps prevent a breakdown by enzymes while targeting cancer-related genes in lung cells and lowering harmful effects on organs, like the kidneys and liver—to put it simply, this helps protect the genetic material and make treatments more precise. Though these developments show NDDS’s possibilities, real-world medical use still faces hurdles, including quality assurance during large-scale manufacturing, long-term safety of material components, and variations in patients’ biological environments. Future studies should prioritize creating smart delivery systems that can adapt to different conditions while also considering how antioxidant substances might address cellular stress caused by environmental particles [223,224].

In addition, the drug delivery technology of metal–organic frameworks (MOFs) is undergoing a rapid evolution from basic drug loading to precise targeting. Studies have shown that the π-π stacking effect of Zr-MOFs can achieve an efficient loading of paclitaxel at 38.7 wt%, and its tumor microacid-triggered release characteristic (>85%) lays the foundation for targeted delivery [225]. Based on this mechanism, by constructing ZIF-8@chitosan capsules, the bottleneck of oral delivery was broken through—chitosan resists gastric acid erosion while MOFs respond to bacterial enzyme release in the colon to release resveratrol, increasing the local drug concentration by 7.2 times and simultaneously enhancing the intestinal barrier permeability by 40% [226]. For more complex brain delivery challenges, experiments have proved that it is possible to further integrate exosome membranes with MIL-101(Fe) to innovatively achieve blood–brain barrier penetration. The targeted modification of neurons enables the accumulation of paclitaxel in the brain to reach 9.3 times that of traditional preparations, and the synergistic treatment catalyzed by the Fe^3+^/Fe^2+^ cycle increases the apoptosis rate of glioma cells to 68.5% [227].

### 7.3. Mechanistic Studies on Antioxidants

Conducting mechanistic studies to explore how specific antioxidants interact with biologically relevant targets in the context of microplastic exposure will provide valuable insights into effective therapeutic strategies [228]. The research on the antioxidant intervention mechanism against microplastic (MP) exposure reveals that melatonin enhances the Nrf2 pathway by competitively binding to Keap1, significantly increasing the activities of SOD and CAT in the liver (MDA decreased by 38% under PE-MPs exposure), while polyphenolic substances (such as resveratrol) inhibit the TLR4/MyD88/NF-κB inflammatory cascade, reducing the expression of IL-6 and TNF-α in intestinal tissues by 50% [59,229,230]. However, MPs have dual interference on the pharmacokinetics of antioxidants. On the one hand, they physically adsorb water-soluble molecules, such as vitamin C (adsorption rate in vitro 40%); on the other hand, they activate CYP450 enzymes to accelerate metabolism and clearance, resulting in a sudden drop in bioavailability [231,232]. What is more complex is that PS-NPs as carriers can increase the intestinal permeability of quercetin by 1.8 times, but they also synergistically induce DNA damage in renal tubules (γ-H2AX ↑ 60%), highlighting the challenge of the toxicity–efficacy balance [7,233]. The breakthrough progress lies in the following. ① The MOF nanodelivery system increases the liver accumulation efficiency of melatonin by 4.8 times and extends the sustained-release duration to 72 h. ② Probiotic-derived butyric acid repairs the intestinal barrier and increases the absorption rate of polyphenols by 55% by upregulating the occludin protein [234,235]. Future research urgently needs to focus on the spatiotemporal dynamics of microplastic–antioxidant complexes in the intestinal–liver axis circulation, the intergenerational effect of Nrf2 epigenetic regulation, and the biomimetic delivery strategy targeting the blood–brain barrier [236,237,238].

### 7.4. Interdisciplinary Approaches

An interdisciplinary approach that combines insights from environmental science, toxicology, nutrition, and public health can foster a holistic understanding of the impacts of microplastics and the role of antioxidants. Collaborations among these fields can enrich research outcomes and enhance intervention strategies.

## 8. Conclusions

Microplastics (MPs) include both primary (such as microparticles from cosmetics) and secondary (degraded fragments of plastics), which accumulate systemically in organs such as the lungs, liver, and intestines through dietary intake, breathing, and skin contact. The topic of reducing the harm of microplastics to human health can be explored through both in vitro research methods (such as advanced biodegradable materials, enzymatic degradation, and nano-enhanced wastewater treatment) and in vivo research methods (such as using antioxidants to mitigate the damage caused by microplastics to the human body). The health risks are mainly mediated by oxidative stress cascade reactions. Mechanistic studies have shown that MPs damage mitochondrial electron transport, induce endoplasmic reticulum stress, activate phagocyte inflammatory responses, and jointly lead to excessive reactive oxygen species (ROS), breaking through the cellular antioxidant defense. Such redox imbalance triggers lipid peroxidation, protein carbonylation, and DNA damage (biomarker changes: MDA↑, 8-OHdG↑, GSH↓) and simultaneously activates the NF-κB pathway to release pro-inflammatory factors, forming the “oxidative-inflammatory vicious cycle axis”.

Intervention experiments have confirmed that natural antioxidants counteract damage through five pathways: (1) directly eliminating ROS (such as ascorbic acid); (2) activating the Nrf2/ARE pathway to enhance the activity of enzymes, such as SOD/CAT (curcumin); (3) promoting mitochondrial biosynthesis (resveratrol); (4) inhibiting NF-κB transcription (quercetin); and (5) regulating autophagy (hesperidin). Empirical cases include astaxanthin, restoring the intestinal barrier function of mice exposed to MPs, and resveratrol, achieving neuroprotection through the SIRT1 pathway. Combined intervention regimens (such as vitamin E + selenium) exhibit synergistic and additive effects.

However, clinical translation faces four major bottlenecks: (i) low oral bioavailability requires delivery via nanocarriers (such as curcumin nanoparticles); (ii) long-term safety is unknown; (iii) there are complex interactions of combined pollutant exposure; and (iv) there is a lack of population epidemiological evidence.

Future research should focus on (1) using multi-omics techniques to analyze the ROS signaling network; (2) developing targeted antioxidant delivery systems; (3) constructing integrated risk assessment standards for environmental toxicology and nutritional epidemiology; and (4) implementing policy interventions that coordinate with microplastic control through dietary guidelines.

Only through interdisciplinary collaborative efforts can we effectively curb the persistent health threat posed by MP pollution.

## Figures and Tables

**Table 1:** Summary of the impact of microplastics on human health.

Impact Category	Key Findings	Target Organs/Systems	References
Oxidative Stress	- Aged PS-MPs loaded with BaP induce colonic barrier damage via oxidative stress-mediated Notch signaling. - PS-MPs trigger lipid peroxidation (↑MDA) and suppress antioxidant enzymes (↓SOD, ↓GSH) in liver organoids.	Colon, Liver	[27,28]
Toxicological Effects	- PS-MPs disrupt hepatic lipid metabolism (↑triglycerides, ↑FASN expression) in liver organoids. - In vitro pulmonary toxicity induces apoptosis and DNA damage in alveolar cells. - Renal tubular injury is linked to MP deposition.	Liver, Lungs, Kidneys	[28,33,35]
Inflammation	↑ Pro-inflammatory cytokines (IL-6, TNF-α) and ↓ goblet cells in colon tissue. - Pulmonary inflammation: Macrophage infiltration and ↑IL-1β/IL-8 in alveoli. - Glomerular inflammation and fibrosis in the kidneys.	Colon, Lungs, Kidneys	[27,33,35]
Bioaccumulation	- MPs detected in human brains (polypropylene/polyethylene via Py-GC/MS). - Deposition in kidneys, blood, and placental tissue confirmed. - Food chain transmission via soil–crop systems.	Brain, Kidneys, Blood, Multi-organ	[26,31,33,36]

↑ indicates that a certain substance is upregulated or increased. ↓ indicates that a certain substance is downregulated or reduced.

**Table 2:** Summary of the classification and mechanism of action of various antioxidants.

Category	Representative Compounds	Mechanism of Action	References
Enzymatic Antioxidants	Superoxide Dismutase (SOD)	Catalyzes the conversion of superoxide radicals (O_2_^−^) → H_2_O_2_ + O_2_ - Maintains ROS dynamic equilibrium - Regulates redox signaling pathways (NF-κB, MAPK, PI3K/Akt)	[93,94,97]
	Catalase (CAT)	Decomposes H_2_O_2_ → H_2_O + O_2_; Prevents hydroxyl radical formation	[14,95,96]
	Glutathione Peroxidase (GPx)	Reduces lipid hydroperoxides and H_2_O_2_ using glutathione (GSH) as an electron donor	[14,95,96]
Non-Enzymatic Antioxidants	Vitamin C, Vitamin E	Direct free radical scavenging - Terminates lipid peroxidation chains (vitamin E) - Regenerates other antioxidants (vitamin C)	[98,99,100]
	Carotenoids	Quenches singlet oxygen and scavenges radicals - Protects eyes/skin - Reduces cardiovascular disease and cancer risk	[98,99,101]
	Flavonoids, Polyphenols	Neutralize radicals via phenolic hydroxyl groups - Chelate metal ions to inhibit ROS generation - Activate endogenous antioxidant enzymes	[98,99,100]

**Table 3:** Conclusions of the differences of sources, bioavailability, dose–effects, and limitations of different antioxidants.

Antioxidant	Sources	Bioavailability	Dose–Effect	Limitations	Mechanisms	In Vitro Evidence	In Vivo Evidence	References
Vitamin C	Citrus fruits, leafy greens	High (water soluble, readily absorbed)	- In vitro effective dose: Significantly reduces ROS, improves cell proliferation - High doses may cause gastrointestinal discomfort	- Short half-life, requiring frequent supplementation - High doses may lead to osmotic diarrhea or kidney stone risk	- Direct ROS scavenging - Inhibits p16/p21 senescence pathway - Reduces lipid peroxidation	- Reduced ROS and SA-β-gal in lung fibroblasts - Inhibited PM-induced senescence - Scavenges ROS in cell-free systems (ORAC assay) - Protects erythrocytes from hemolysis induced by oxidative stress	- Lowered AAC risk via oxidative stress reduction - Synergizes with selenium to reduce hepatic oxidative damage in rats - Enhances recovery from MP-induced inflammation in zebrafish	[133,134,135,157,158]
Curcumin	Turmeric rhizomes	Low (lipophilic, requires lipid co-ingestion)	- In vitro effective dose: Activates the Nrf2 pathway, enhances antioxidant enzymes - Requires high doses in animal models	- Poor bioavailability, requires nanoformulations or piperine co-administration - Long-term high doses may impair iron absorption	- Activates Nrf2-Keap1 pathway - Upregulates HO-1, SOD, CAT - Scavenges ROS	- Improved cell viability and antioxidant enzymes in RAW 264.7 cells - Activates Nrf2/ARE pathway in human keratinocytes - Inhibits NF-κB-mediated pro-inflammatory cytokine release	- Attenuated oxidative stress in animal models - Attenuates MP-induced liver fibrosis in mice via TGF-β suppression - Improves cognitive function in MP-exposed rats	[111,136,159,160,161]
Quercetin (QSN)	Apples, onions, tea (nanoformulations)	Moderate (nanoformulations improve targeting)	- Nanoformulations target intestinal tissues, reduce TNF-α/IL-6 - Anticancer effects require dose validation	- Low absorption in non-nano forms - Nanoformulations may have unknown toxicity or metabolic issues	- Inhibits NF-κB/MAPK pathways - Scavenges ROS - Anti-inflammatory (↓TNF-α, IL-6)	- Reduced oxidative stress in intestinal cells - Anticancer effects via cell cycle regulation - Nano-encapsulation improves uptake in Caco-2 intestinal cells - Chelates Fe^2+^ to prevent hydroxyl radical formation	- Limited direct in vivo microplastic studies; general antioxidant effects shown - Reduces MP-induced gut microbiota dysbiosis in zebrafish - Protects against renal oxidative stress (↓ MDA, ↑ SOD)	[126,137,138,162]
Resveratrol	Grapes, red wine, nuts	Low (rapid metabolism, significant first-pass effect)	- Effective in vitro/in vivo: Reduces ROS, activates SIRT1/Nrf2 pathways - Requires high doses for sustained effects	- Poor bioavailability, needs lipid carriers or sustained-release formulations - High doses may inhibit CYP450 enzyme activity	- Activates SIRT1/Nrf2 - Inhibits NF-κB - Improves mitochondrial function	- Reduced ROS and ↑HO-1 in cell models - Upregulates SIRT1, enhancing mitochondrial biogenesis in SH-SY5Y cells - Suppresses NLRP3 inflammasome activation	- Alleviated neuroinflammation in mice - Protected against liver/kidney damage - Extends lifespan in C. elegans exposed to MPs - Improves endothelial function in hypertensive rats	[139,140,150,151]
Melatonin (MT)	Plants	High bioavailability in plants; efficiently absorbed and distributed in tissues Efficiently absorbed and distributed in tissues	- Dose-dependent enhancement of stress tolerance and antioxidant activity	- -Optimal dosage varies among species - -Excessive use may disrupt endogenous hormone balance	- Direct ROS scavenging - Upregulates antioxidant enzymes - Modulates Ca^2+^/MAPK signaling	- Enhanced stress tolerance in plants - Directly scavenges ·OH and ONOO^−^ in neuronal cells - Modulates epigenetic markers (DNA methylation) in astrocytes	- Protected adrenal cortex in rats - Improved mitochondrial function - Crosses BBB to reduce neuroinflammation in MP-exposed mice - Mitigates MP toxicity in agricultural plants	[58,142,143,144,163]
Glutathione (GlyNAC)	Endogenous (glycine + NAC precursors)	Limited oral absorption (requires precursor supplementation)	- GlyNAC restores mitochondrial membrane potential, increases ATP - Promotes mitophagy, reduces DNA damage	- Low oral GSH absorption - Long-term combination safety is unclear	- ↑GSH levels - Restores mitochondrial function (↑ATP, ↓ROS) - Promotes mitophagy	- Limited direct microplastic studies - Restores mitochondrial GSH levels in HepG2 cells - Binds to MP surfaces, reducing cellular uptake	- Reduced liver oxidative stress in rats - Improved lifespan via mitochondrial repair - Reverses age-related glutathione depletion in mouse models - Synergizes with NAC to protect lung tissue	[148,149,164]
Astaxanthin	Algae, salmon, krill	Moderate (lipophilic, requires dietary fats)	- Animal model effective dose: Activates SIRT1, inhibits NF-κB - Improves mitochondrial function and histopathology	- High doses are costly - Natural sources are limited; synthetic variants may differ in activity	- Activates SIRT1 - Inhibits NF-κB - Improves mitochondrial function	- Reduced ferroptosis in chondrocytes - 10× stronger ROS scavenging than vitamin E (ORAC assay) - Protects HaCaT cells from UV-MP co-exposure	- Protected fish from microplastic-induced stress - Attenuated osteoporosis in mice - Reduces skin photoaging in MP-fed mice - Enhances stress tolerance in aquaculture species	[152,153,154,165,166,167]
Beta-carotene	Carrots, leafy greens	Dependent on dietary fats (variable conversion efficiency)	- Enhances SOD/CAT/GPx activity - Anticancer effects require dose validation (e.g., apoptosis induction)	- High doses may increase lung cancer risk in smokers - Excess causes carotenodermia (skin yellowing)	- ↑SOD, CAT, GPx - Induces apoptosis in cancer cells	- Enhanced antioxidant enzymes in cell models - Inhibits lipid peroxidation in liposome models - Downregulates COX-2 in RAW 264.7 macrophages	- Mitigated copper-induced stress in zebrafish - Prevents retinal degeneration in quail exposed to MPs - Reduces breast cancer incidence in chronic MP exposure models	[155,156,168,169]
Combination Therapy (e.g., Vitamin E + Selenium)	Nuts, fish, whole grains	Synergistic effects (absorption mechanisms require optimization)	- Preliminary synergy in cytoprotection (e.g., reduced oxidative damage) - Optimal ratios/doses need further study	- Complex multi-component interactions - Long-term use may cause antagonism or toxicity	- Vitamin E interrupts lipid peroxidation - Se supports GPx activity - Regenerates oxidized vitamin E	- Enhanced cytoprotection in cellular models - Selenium-dependent GPx4 activation in enterocytes - Vitamin E regenerates oxidized selenoproteins	- Ameliorated BPA toxicity in rats - Reduces MP-induced nephrotoxicity in rats (↓ KIM-1) - Improves soil microbial health in MP-contaminated fields	[102,103,147,157]

↑ indicates that a certain substance is upregulated or increased. ↓ indicates that a certain substance is downregulated or reduced.

## Data Availability

No new data were created or analyzed in this study.

## Abbreviations

The following abbreviations are used in this manuscript:

ROS	Reactive oxygen species
SOD	Superoxide dismutase
GPx	Glutathione peroxidase
PVC	Polyvinyl chloride
PS	Polystyrene
PLA	Polylactide acid
Th2	T-helper type 2
MPs	Microplastics
NPs	Nanoplastics
ETC	Electron transport chain
mPTP	Mitochondrial permeability transition pore
BPA	Bisphenol A
MDA	Malondialdehyde
BER	Base excision repair
NER	Nucleotide excision repair
PS-MPs	Polystyrene microplastics
PE-NPs	Polyethylene nanoplastics
ER	Endoplasmic reticulum
UPR	Unfolded protein response
miRNAs	MicroRNAs
GSH	Glutathione
PUFAs	Polyunsaturated fatty acids
ARE	Antioxidant response element
SFN	Sulforaphane
MMP	Mitochondrial membrane potential
AST	Astaxanthin
ALT	Alanine aminotransferase
I/R	Ischemia/reperfusion
GST	Glutathione S-transferase
T2D	Type 2 diabetes
AAC	Abdominal aortic calcification
QSN	Quercetin
MT	Melatonin
PE-MPs	Polyethylene microplastics
NAC	N-acetylcysteine
Gly	Glutathione
SIR 2.1	Sirtuin 2.1
HLH-30	Helix–loop–helix 30
PP-MPs	Polypropylene microplastics
CoQ10	Coenzyme Q10
